# Time-averaged serum potassium levels and its fluctuation associate with 5-year survival of peritoneal dialysis patients: two-center based study

**DOI:** 10.1038/srep15743

**Published:** 2015-10-28

**Authors:** Shen-Heng Li, Jian-Teng Xie, Hai-Bo Long, Jun Zhang, Wei-Dong Zhou, Hong-Xin Niu, Xun Tang, Zhong-Lin Feng, Zhi-Ming Ye, Yang-Yang Zuo, Lei Fu, Feng Wen, Li-Ping Wang, Wen-Jian Wang, Wei Shi

**Affiliations:** 1 Division of Nephrology, Zhujiang Hospital, Southern Medical University, Guangzhou, 510282, China; 2 Division of Nephrology, Guangdong General Hospital, Guangdong Academy of Medical science, Guangzhou, 510080, China

## Abstract

The time-averaged serum potassium was more comprehensive to reflect the all-time changes of serum potassium levels during peritoneal dialysis (PD). However, the association of fluctuation of time-averaged serum potassium level with long-time survival of PD patients remains unknown. In this retrospective study, we included 357 incident PD patients in 2 centers from January 1, 2007 to October 31, 2012 with follow-up through October 31, 2014. Our data demonstrated that it was the lower time-averaged serum potassium level rather than baseline of serum potassium level that was associated with high risk of death. Patients with higher standard deviation (SD) had significantly poorer all-cause (*p* = 0.016) and cardiovascular mortality (*p* = 0.041). Among the patients with time-averaged serum potassium levels below 4.0 mEq/L, a lower mean value was more important than its SD to predict death risk. In contrast, the patients with time-averaged serum potassium levels above 4.0 mEq/L, those with serum potassium SD < 0.54 mEq/L, exhibited a higher 3-year and 5-year survival rate for both all-cause and cardiovascular mortality compared to the control groups. Our data clearly suggested both time-averaged serum potassium and its fluctuation contributed disproportionately to the high death risk in PD patients.

Hypokalemia is the most common electrolyte disorder in patients on PD^1^. Single-center studies from Taiwan (n = 140) and Hong Kong (n = 266) have shown an association between hypokalemia in PD patients and Enterobacteriaceae peritonitis and all-cause mortality, respectively^2^,^3^. A large peritoneal dialysis cohort study showed that PD patients were more likely to have serum potassium <4.0 mEq/L, with an adjusted odds ratio of 3.30 (95% confidence interval [CI], 3.05−3.56)^4^,^5^. A U-shaped relationship between time-averaged serum potassium and all-cause and cardiovascular mortality of PD patients was demonstrated^4^,^5^. A recent study found that patients with a relative stable serum potassium levels during the first year observation appeared to have a pronouncedly better survival, an excessive fluctuation of serum potassium was associated with significantly increased mortality, which was independent of the average serum potassium levels^6^. The time-averaged serum potassium was obtained for up to 20 calendar quarters to calculate^4^, and was more comprehensive to reflect the overall changes of serum potassium levels combined with its fluctuation. However, the association of SD of the time-averaged serum potassium with the long time survival of PD patients remains unknown. Current study is designed to evaluate the relations of time-averaged serum potassium level and its SD and the 5-year survival of PD patients in 2 centers.

## Results

### Incidence of serum potassium abnormalities

Table 1 showed the baseline demographics of the included patients in 2 centers. Distribution of serum potassium levels and corresponding mortality rates were presented in Table 2. We found a 19.3% incidence of hypokalemia by time-averaged serum potassium and a 24.4% incidence of hypokalemia by baseline serum potassium, which was similar to a previous reported 27.9% in China^6^. As shown in Table 3, the patients with time-averaged serum potassium levels <4.0 mEq/L exhibited a significant higher adjusted HR for all-cause mortality compared to those ≥4.0 mEq/L. However, there was no significant difference of adjusted HR for cardiovascular mortality (*p* = 0.225, adjusted HR 1.501, 95% CI, 0.779−2.892) between the groups.

### Time-averaged serum potassium levels rather than baseline serum potassium levels associated with PD patient’s survival

**Figure 1:**
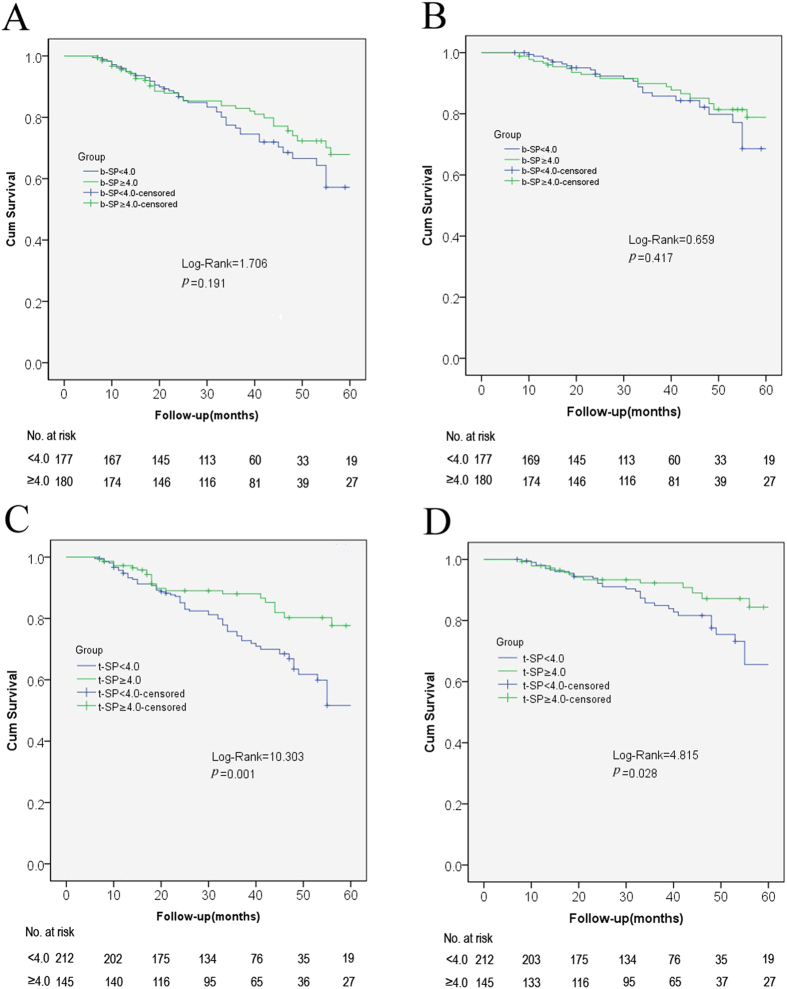
*Kaplan-Meier* survival curves for mortality according to baseline and time-averaged serum potassium. All-cause mortality (**A**) and cardiovascular mortality (**B**) based on baseline serum potassium(mEq/L); (**C,D**). All-cause mortality and cardiovascular mortality based on time-averaged serum potassium(mEq/L).

*Kaplan-Meier* survival curves for mortality according to baseline and time-averaged serum potassium were presented in Fig. 1. There were no significant differences of cumulative survival between the patients with serum potassium <4.0 mEq/L and those ≥4.0 mEq/L according to baseline serum potassium level (Fig. 1A,B). But the patients with time- averaged serum potassium levels above 4.0 mEq/L exhibited a higher cumulative survival for both all-cause mortality (Fig. 1C) and cardiovascular mortality (Fig. 1D) compared to those below 4.0 mEq/L.

### Association of mortality and time-averaged serum potassium fluctuation

**Figure 2:**
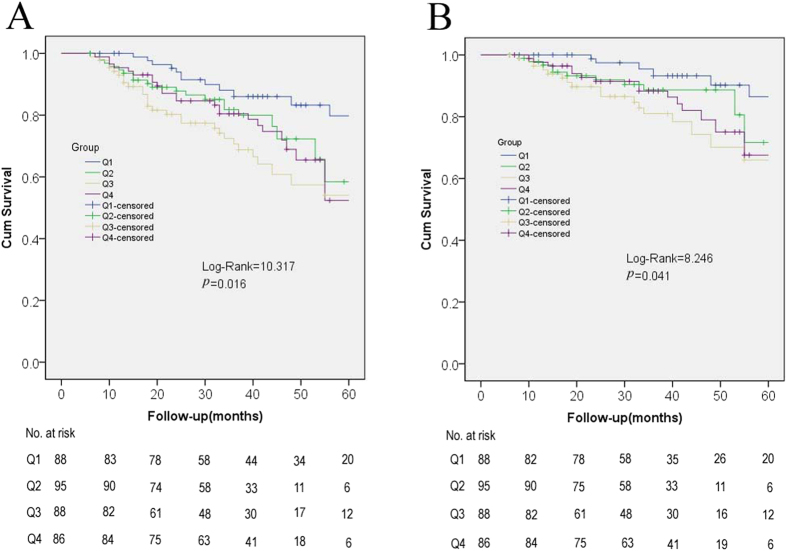
*Kaplan-Meier* survival curves for mortality according to time- averaged serum potassium fluctuation All-cause mortality (A); Cardiovascular mortality (B). Patients were categorized into four groups according to quartiles (Q) of standard deviation of time-averaged serum potassium: Q1: <0.39 mEq/L; Q2: 0.39 mEq/L to <0.52 mEq/L; Q3: 0.52 mEq/L to <0.69 mEq/L; Q4: ≥0.69 mEq/L. There was no difference of the levels of time-averaged serum potassium among the 4 SD groups (*p* = 0.49, Table 4).

**Figure 3:**
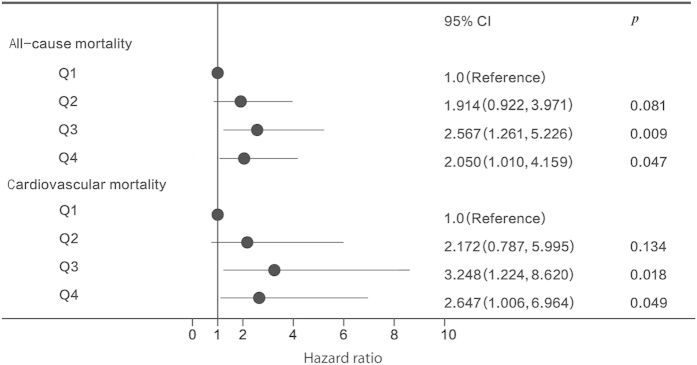
Hazard ratios for all-cause and cardiovascular mortality according to time-averaged serum potassium fluctuation. Q1: <0.39 mEq/L; Q2: 0.39 mEq/L to <0.52 mEq/L; Q3: 0.52 mEq/L to <0.69 mEq/L; Q4: ≥0.69 mEq/L.

Patients were categorized into four groups according to the quartiles (Q) of SD of time-averaged serum potassium: Q1: <0.39 mEq/L; Q2: 0.39 mEq/L to <0.52 mEq/L; Q3: 0.52 mEq/L to <0.69 mEq/L; Q4: ≥0.69 mEq/L. Table 4 showed baseline characteristics of the 4 groups. There was no significantly difference of most clinical characteristics between groups. *Kaplan-Meier* survival curves and adjusted HR were presented in Figs 2 and 3. Our data indicated that patients with higher SD exhibited significantly poorer all-cause (*p* = 0.016) and cardiovascular mortality *(p* = 0.041), compared to those with relatively low SD. Interestingly, patients from Q3 group rather than Q4 group demonstrated the highest death risk. Patients from Q2 group also exhibited a tendency of high death risk, although the difference was not significant, all-cause (*p* = 0.081, adjusted HR 1.914, 95%CI, 0.922−3.971) and cardiovascular mortality (*p* = 0.134, adjusted HR 2.172, 95% CI, 0.787−5.995). Table 5 displayed logistic regression analysis of risk factors for increased serum potassium fluctuation (SD ≥ 0.39 mEq/L) based on time-averaged serum potassium, and these factors were used to adjust for all-cause and cardiovascular mortality in this study, which is consistent with previous studies^4^,^6^.

### Association of mortality and serum potassium fluctuation in the patients with different range of time-averaged serum potassium levels

**Figure 4:**
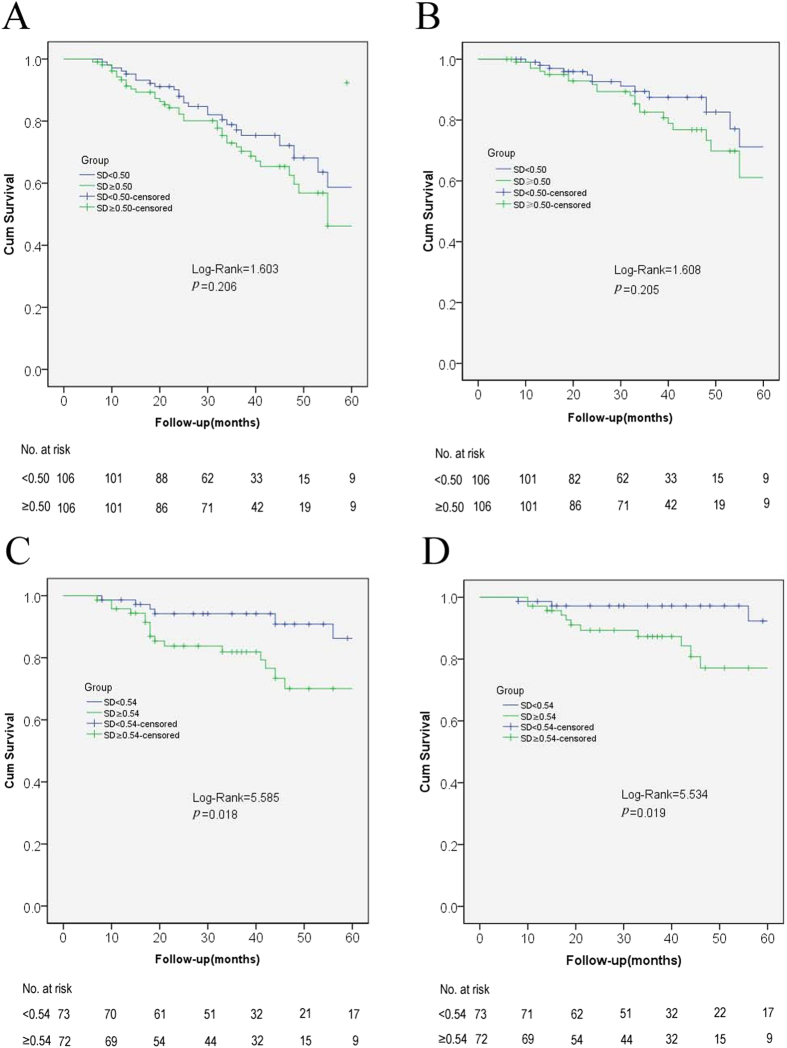
*Kaplan-Meier* survival curves for mortality according to standard deviation in different range of time-averaged serum potassium levels. All-cause mortality (**A**) and cardiovascular mortality (**B**) based on potassium fluctuation in patients with time-averaged serum <4.0 mEq/L. The median of SD was 0.50 mEq/L, and there was no difference of the levels of time-averaged serum potassium between the 2 SD groups (*p* = 0.88); All-cause mortality (**C**) and cardiovascular mortality (**D**) based on potassium fluctuation in patients with time-averaged serum ≥4.0 mEq/L. The median of SD was 0.54 mEq/L, and there was no difference of the levels of time-averaged serum potassium between the 2 SD groups (*p* = 0.66).

*Kaplan-Meier* survival curves for mortality according to time-averaged serum potassium fluctuation in different range of time-averaged serum potassium levels were shown in Fig. 4. Among the patients with time-averaged serum potassium levels below 4.0 mEq/L, there was no significant difference for both all-cause mortality (Fig. 4A) and cardiovascular mortality (Fig. 4B), between the groups of different fluctuation indicating that the time-averaged serum potassium level is more important to predict the death risk than its fluctuation in this population. In contrast, among the patients with time-averaged serum potassium levels above 4.0 mEq/L, patients with a larger fluctuation seemed to face a greater risk of all-cause and cardiovascular mortality (Fig. 4C,D and Table 3).

### Survival rate by time-averaged serum potassium and its fluctuation

As shown in Table 6, the patients with time-averaged serum potassium ≥4.0  mEq/L exhibited a higher 5-year survival rate compared to those <4.0 mEq/L for both all-cause and cardiovascular mortality. Further more, among the groups of time-averaged serum potassium ≥4.0 mEq/L, the patients with serum potassium SD <0.54 mEq/L exhibited a higher 3-year and a 5-year survival rate for both all-cause and cardiovascular mortality, compared to the those ≥0.54 mEq/L.

## Discussion

In the present study, our data clearly demonstrated that time-averaged serum potassium was reliable and comprehensive to evaluate the poor outcome of hypokalemia in PD patients. We found that it was a low time-averaged serum potassium level rather than a low baseline of serum potassium level that was associated with death risk. Importantly, among the patients with time-averaged serum potassium levels below 4.0 mEq/L, a lower mean value was more important than its SD to predict death risk. In contrast, the patients with time-averaged serum potassium levels above 4.0 mEq/L, those with serum potassium SD below 0.54 mEq/L, exhibited a higher 3-year and 5-year survival rate for both all-cause and cardiovascular mortality than others.

Time-averaged serum potassium was obtained from all-time changes of serum potassium levels, and was more comprehensive to reflect serum potassium level compared to the baseline serum potassium. It has been reported that the incidence of hypokalemia is 10% to 60% in patients on PD when hypokalemia is defined as serum potassium level of less than 3.5 mEq/L or requirement of potassium supplement^3^,^4^,^6^,^7^,^8^,^9^,^10^. In the current study, we reported that 19.3% of Chinese PD patients presented time-averaged serum potassium <3.5 mEq/L and 24.4% patients presented baseline serum potassium <3.5 mEq/L. Although the incidence of hypokalemia were almost same, we reported a 59.4% incidence of time-averaged serum potassium <4.0 mEq/L, which is consistent with the previous large cohort study that PD patients were significantly more likely to have time-averaged serum potassium <4.0 mEq/L^4^. Patients with hypokalemia exposed not only to the dialysate potassium loss but also the presence of other factors such as inadequate dietary intakes and/or transcellular shifts and drug taking^11^,^12^,^13^. A high frequency monitoring is helpful to find hypokalemia and give a potassium-supplement immediately, thus reduces the time to expose to such a condition. In the current study, we found that time-averaged serum potassium levels, rather than baseline serum potassium levels, associated with PD patient’s survival.

Although hypokalemia is usually defined as serum potassium level of less than 3.5 mEq/L, serum potassium levels of 3.5 to <4.0 mEq/L was associated with a higher death risk compared with serum potassium levels of 4.0 to <4.5 mEq/L. A U-shaped relationship between time-averaged serum potassium and all-cause and cardiovascular mortality in PD-treated patients had been demonstrated, further more, the population-attributable risk for deaths with serum potassium <4.0 mEq/L was almost two-fold higher than seen with levels ≥5.5 mEq/L in PD patients^4^. Therefore, we evaluated the death risk of time-averaged serum potassium based on the cut-point of 4.0 mEq/L in present study.

Serum potassium abnormalities most prominently affect the cardiovascular system and have been implicated in many aspects of cardiovascular disease including atrial fibrillation, stroke, heart attack, hypertension, sudden cardiac death^14^ as well as other risk factors^15^,^16^,^17^,^18^, and many studies focused on the death risk of serum potassium levels rather than its fluctuation. Our study displayed that a lower mean value was more important than its SD to predict death risk in the patients with time-averaged serum potassium levels below 4.0 mEq/L. However, among the patients with time-averaged serum potassium levels above 4.0 mEq/L, the patients with serum potassium SD <0.54 mEq/L exhibited a higher 3-year and a 5-year survival rate for both all-cause and cardiovascular mortality, compared to those ≥0.54 mEq/L. Consistently, patients with a relative stable time-averaged serum potassium level appeared to have a pronouncedly better survival, an excessive fluctuation in time-averaged serum potassium was associated with significantly increased all-cause (*p* = 0.016) and cardiovascular mortality (*p* = 0.041), which was independent of the time-averaged serum potassium levels. A recently single-center study of Chinese patients demonstrated a higher risk for all-cause mortality and cardiovascular mortality in PD patients with fluctuation of serum potassium^6^, but, only the first year’s fluctuation was analyzed. Thus, our findings build up on and extend those from the previous report. In our study, it was the moderate increased SD that was associated with the highest death risk, indicating that a dangerous range of SD based on time-averaged serum potassium were associated with significant death risk, and both time-averaged serum potassium and its fluctuation contributed disproportionately to the high death risk in PD patients.

Previous studies reported that hypokalemia was outward manifestation of hypoalbuminemia and malnutrition^2^,^3^,^4^,^6^,^19^, which are the key important factors for the long-term patient survival^3^,^20^,^21^,^22^. Time-averaged serum potassium level is a more meaningful parameter to evaluate the nutritional status from this standpoint. Our study showed nutritive indexes including BMI and albumin among different SD groups had significant difference (*p* = 0.04, *p* = 0.01 respectively), and multivariate logistic regression showed that both BMI and albumin were independent factors associated with serum potassium fluctuation. So our findings emphasized the importance of time-averaged serum potassium associated with its fluctuation as a nutritional marker as well as potential predictor of outcome. Importantly, hypokalemia is also a surrogate marker of severe comorbid illness^3^, which is associated with high risk of death in PD patients^23^. Consistent with this opinions, we found that there were significant differences of CCI between different SD groups (*p* = 0.001), and CCI was an independent factor associated with serum potassium fluctuation.

The benefit of potassium supplementation in hypokalemic patients has not been established. Our data suggested that it seemed prudent for nephrologists to monitor serum potassium level frequently and keep potassium homeostasis stable to reduce the fluctuation of serum potassium in patients on peritoneal dialysis. Monitoring serum potassium every month is effective to find the patients with serum potassium abnormalities timely during the course of PD modality.

Nonetheless, our study has several limitations. First, this retrospective study included only two PD centers from one district in China, and the sample size is small. Thus, the selection bias would be inevitable, and when we evaluated the association between serum potassium fluctuation and mortality, only 4.0% of patients had time-averaged serum potassium ≥5.0 mEq/L, which is lower than previous report^4^. Second, although we are certain that a low time-averaged serum potassium level and an excessive fluctuation was the indicator of poor prognosis, it may be a surrogate marker of an underlying cause of excess mortality, as we previous discussed. Third, factors affecting serum potassium levels, such as dietary intake, loss via dialysate and urine, were not examined in the present study.

In conclusion, our study demonstrates for the first time that both time-averaged serum potassium and its excessive fluctuation contributed disproportionately to the high death risk in PD patients. Our findings emphasize the importance of time-averaged serum potassium which was obtained with high frequent monitoring and was crucial to keep potassium homeostasis stable during all time of PD-treatment.

## Methods

### Patients and study design

A total of 475 incident CAPD patients with 18 years or older (18–81 years) and being treated with PD at Guangdong General Hospital, the Peritoneal Dialysis Quality Control Center of Guangdong Province, and Zhujiang Hospital of Southern Medical University from January 1, 2007 to October 31, 2012 with follow-up through October 2014 were studied. All patients were treated with Dianeal solution which does not contain potassium (Baxter China Ltd., Guangzhou, China). Patients were categorized according to baseline serum potassium levels (<4.0 mEq/L, ≥4.0 mEq/L) and time-averaged serum potassium levels (<4.0 mEq/L, ≥4.0 mEq/L) to examine the association between serum potassium and all-cause and cardiovascular mortality. For each patient the baseline data was defined as the average of all repeated measures done during the first 3 months^4^. Patients surviving more than 6 months with at least 3 check-ups for serum potassium were selected to investigate the relation of serum potassium variability and mortality, similar to the previous reports^6^. The maximum number of repeated measurement of serum potassium was 24, with an average follow-up time of 36.9 ± 17.6 months. The time-averaged serum potassium was obtained for up to 24 calendar quarters to calculate the time-averaged serum potassium. We excluded 37 patients who commenced PD for less than 6 months, because one third of all PD failures and 40% of all technique failures may occur within the first 6 months^24^. We also excluded another 81 patients due to following reasons: transferring to hemodialysis (HD) (n = 33), renal transplantation (n = 28), and less than three times measurement of serum potassium levels (n = 20). The reason for participants censored at the time of transferring to HD modality or renal transplantation is because that the risk with low time-averaged serum potassium levels decreased to nonsignificant level, whereas the risk with higher serum potassium levels persisted^4^. 357 of 475 patients were included in this analysis, including 193 patients in Zhujiang Hospital of Southern Medical University. Serum potassium fluctuation was expressed as the SD of time-averaged serum potassium. Patients were categorized to four groups according to quartiles (Q) of standard deviation: Q1: <0.39 mEq/L; Q2: 0.39 mEq/L to <0.52 mEq/L; Q3: 0.52 mEq/L to <0.69 mEq/L; Q4: ≥0.69 mEq/L. This study was approved as exempt from informed consent by Medical Ethics Committee of Zhujiang Hospital of Southern Medical University and Guangdong General Hospital, Guangdong Academy of Medical Science respectively. The methods in this study were in accordance with the guidelines of Medical Ethics Committee of the 2 institutions.

### Demographic and clinical data

All data were obtained at the time of PD initiation, including demographic details, etiology of end-stage renal disease (ESRD), presence of diabetes, and comorbidity score. Comorbidity was measured using the modified CCI^23^. Baseline biochemical data, a standard peritoneal equilibration test (PET) and Kt/V was evaluated in the first 3 months of PD therapy, as previous described^25^. Urine and ultrafiltration volumes during Kt/V test^26^, peritoneal transport status (D/PCr), and adequacy of dialysis [total Kt/V_urea_ and total weekly creatinine clearance (WCCr)] were measured using PD Adequest software (Baxter Healthcare Corporation, Chicago, 1L, USA). Residual glomerular filtration rate (rGFR) was calculated as the average of 24-hour urinary urea and creatinine clearance^27^. Daily exchange volume of peritoneal dialysate was defined as peritoneal dialysis volume per unit of body surface area (PDV/BSA)^6^ during the first 3 months of PD treatment. All biochemical and hematological tests were measured in the biochemical laboratory of Zhujiang Hospital of Southern Medical University and Guangdong General Hospital, Guangdong Academy of Medical science. Clinical Test Center of Ministry of Health, China and Clinical Test Center of Guangdong Province are responsible for quality control (External Quality Assessment, EQA) including test of serum potassium in the two centers, three times a year (March, June and September), two times a year (April and August) respectively.

### Detection and management of hypokalemia and hyperkalemia

All patients were follow-up every 3 months with serum potassium measured at each visit, and additional measurement would be determined according to patients’ condition (including vomiting, diarrhea, peritonitis or hospitalization). Serum potassium was measured using an ion-sensitive electrode. Hypokalemia (serum potassium <3.5 mEq/L) or hyperkalemia was informed to the clinics urgently for immediate recheck or treatment. The target of serum potassium level was 3.5 to 5.5 mEq/L. Patients were instructed to have a renal diet low in phosphorus. Patients with normal level of serum potassium did not receive potassium supplement. Patients with hypokalemia were given oral, intravenous, or intraperitoneal potassium supplement according to the serum potassium levels. Patients with serum potassium levels of 3.0 to 3.5 mEq/L, 2.5 to 3.0 mEq/L, and less than 2.5 mEq/L were given oral potassium chloride extended release formulation, oral potassium chloride combined with intraperitoneal potassium supplement via dialysate, and intravenous potassium supplement, respectively. For patients with a potassium level ≥5.5 mEq/L, high potassium diet and hyperkalemia generating drugs (angiotensin converting enzyme inhibitors, angiotension receptor antagonists, and potassium-sparing diuretics) would be stopped immediately, conventional potassium reduction method (including furosemide, 50% glucose plus insulin, sodium bicarbonate or intravenous calcium supplement) was given. Intermittent peritoneal dialysis (IPD) or HD (only 1 patient) was used to remove extra potassium if necessary. Serum potassium levels were then monitored closely and maintained in the normal range (serum potassium concentration of 3.5 to <5.5 mEq/L). Intervention of serum potassium was within a short time by medical routine (about 1−7 days). And serum potassium level was the clinical manifestation of the actual condition of each visit.

### Outcomes

Primary outcomes were all-cause mortality and cardiovascular mortality. Cardiovascular mortality was defined as death from acute heart failure, myocardial infarction, fatal arrhythmia, stroke, peripheral artery disease^28^,^29^, or sudden death. Survival was defined as the time from enrollment to death or administrative censoring (i.e. transfer to other dialysis centers, loss to follow-up, or end of the study period) at October 31, 2014.

### Statistical Analyses

Results are expressed as mean ± SD unless otherwise specified. Comparisons between parameters were performed using *ANOVA*, *Kruskal-Wallis H* test, or *Chi-Square* test, as appropriate. Survival analyses were performed to assess associations of baseline, time-averaged serum potassium levels and standard deviation levels with all-cause mortality and cardiovascular mortality, with the category of lowest death risk group as the reference. The *Cox* models were constructed by adjusted for age, gender, BMI, diabetic status, CCI, hemoglobin, serum albumin level, and high-sensitivity reactive protein (Hs-CRP), because these parameters were related to serum potassium levels and/or significantly associated with mortality in univariate *Cox* proportional hazard regression analyses in previous studies^4^,^6^, or with risk of increased serum potassium fluctuation in univariate or multivariate logistic regression analyses in this study. The prognostic significance of time-averaged serum potassium and its fluctuation was determined by means of *Kaplan-Meier* survival curve and *Cox* proportional hazards regression model. All statistical analyses were performed using the *SPSS* version 16.0 (SPSS Inc., Chicago, IL, USA). A *p*-value < 0.05 was considered statistically significant.

## Additional Information

**How to cite this article**: Li, S.-H. *et al.* Time-averaged serum potassium levels and its fluctuation associate with 5-year survival of peritoneal dialysis patients: two-center based study. *Sci. Rep.* **5**, 15743; doi: 10.1038/srep15743 (2015).

## Figures and Tables

**Table 1:** Baseline characteristics of peritoneal dialysis patients in the 2 centers.

	GuangdongGeneral Hospital(n = 164)	ZhujiangHospital(n = 193)
Female, n (%)	77 (46.9)	86 (44.6)
Age, years	49.5 ± 16.3	50.9 ± 15.1
Etiology of ESRD		
CGN, n (%)	76 (46.3)	93 (48.2)
DN, n (%)	27 (16.5)	27 (14.0)
HN, n (%) (%)	38 (23.2)	45 (23.3)
Others, n (%)	23 (14.0)	28 (14.5)
Diabetes, n (%)	30 (18.3)	32 (16.6)
Use of ACEI/ARB at study initiation, n (%)	75 (45.7)	86 (44.6)
History of loop diuretic, n (%)	24 (14.6)	33 (17.1)
CCI, score	3.63 ± 1.82	3.55 ± 1.42

Data expressed by mean ± SD, number (percent). ESRD, end stage renal disease; CGN, chronic glomerulonephritis; DN, diabetic nephropathy; HN, hypertensive nephrosclerosis; ACEI, angiotensin-converting enzyme inhibitors; ARB, angiotensin II receptor blockades; CCI, Charlson Comorbidity Index.

**Table 2:** Distribution of serum potassium levels and corresponding mortality rates.

		Incidence of abnormalitiesin serum potassium		Overall deathpercentage ineach group		Cardiovasculardeath percentage ineach group	
Serum potassium (mEq/L)		b-SP (n = 357)	t-SP (n = 357)	b-SP	t-SP	b-SP	t-SP
<4.0	<3.0	31 (8.7)	12 (3.4)	11 (35.5)	5 (41.7)	7 (22.6)	3 (25.0)
	3.0 to <3.5	56 (15.7)	57 (16.0)	11 (19.6)	20 (35.1)	5 (8.9)	9 (15.8)
	3.5 to <4.0	90 (25.2)	143 (40.1)	24 (26.7)	37 (25.9)	14 (15.6)	23 (16.1)
	Total	177 (49.6)	212 (59.4)	46 (26.0)	62 (29.2)	26 (14.7)	35 (16.5)
≥4.0	4.0 to <4.5	102 (28.6)	102 (28.6)	16 (15.7)	14 (13.7)	10 (9.8)	9 (8.8)
	≥4.5	78 (21.8)	43 (12.0)	22 (28.2)	8 (18.6)	13 (16.7)	5 (11.6)
	Total	180 (50.4)	145 (40.6)	38 (21.1)	22 (15.2)	23 (12.8)	14 (9.7)

Data expressed by number (percent). b-SP, Baseline serum potassium. t-SP, Time-averaged serum potassium.

**Table 3:** Hazard ratios for all-cause and cardiovascular mortality according to time-averaged serum potassium levels and its fluctuation.

t-SP	HR	95% CI		*p*value	SD (t-SP≥4.0)	HR	95% CI		*p*value
		Lower	Upper				Lower	Upper	
Crude all-cause mortality									
≥4.0	Ref	Ref	Ref		<0.54	Ref	Ref	Ref	
<4.0	2.173	1.334	3.539	0.002	≥0.54	2.934	1.146	7.510	0.025
aAdjusted all-cause mortality									
≥4.0	Ref	Ref	Ref		<0.54	Ref	Ref	Ref	
<4.0	1.743	1.046	2.906	0.033	≥0.54	2.883	1.092	7.617	0.033
Crude cardiovascular mortality									
≥4.0	Ref	Ref	Ref		<0.54	Ref	Ref	Ref	
<4.0	1.977	1.061	3.682	0.032	≥0.54	4.119	1.146	14.804	0.030
aAdjusted cardiovascular mortality									
≥4.0	Ref	Ref	Ref		<0.54	Ref	Ref	Ref	
<4.0	1.501	0.779	2.892	0.225	≥0.54	3.783	1.038	13.390	0.044

^a^Adjustments included age at initiation of PD, gender, BMI, diabetes status, CCI, hemoglobin, serum albumin and Hs-CRP. t-SP, time-averaged serum potassium (mEq/L). HR, hazard ratio; SD, standard deviation. The median of SD in patients with t-SP ≥ 4.0 mEq/L was 0.54 mEq/L.

**Table 4:** Patient baseline data by quartiles of standard deviation.

Variables	Q1 (n = 88)	Q2 (n = 95)	Q3 (n = 88)	Q4 (n = 86)	*p* value
Time averaged serum potassium, mEq/L	3.90 ± 0.52	3.83 ± 0.48	3.87 ± 0.47	3.94 ± 0.49	0.49
Female, n (%)	39 (44.3)	42 (44.2)	40 (45.4)	42 (48.8)	0.92
Age, years	47.2 ± 13.7	48.3 ± 15.7	51.9 ± 15.1	53.3 ± 15.0	0.02
BMI, kg/m^2^	22.6 ± 3.0	22.5 ± 3.1	21.6 ± 2.6	21.8 ± 2.7	0.04
Etiology of ESRD					
CGN, n (%)	46 (52.3)	38 (40.0)	36 (40.9)	39 (45.3)	0.67
DN, n (%)	10 (11.3)	18 (19.0)	17 (19.3)	9 (10.5)	
HN, n (%)	19 (21.6)	23 (24.2)	20 (22.8)	21 (24.4)	
Others, n (%)	13 (14.8)	16 (16.8)	15 (17.0)	17 (19.8)	
Diabetes, n (%)	11 (12.5)	20 (21.1)	19 (21.6)	12 (14.0)	0.30
Use of ACEI/ARB at study initiation, n (%)	41 (46.6)	40 (42.1)	38 (43.2)	42 (48.8)	0.85
History of loop diuretic, n (%)	16 (18.2)	17 (17.9)	12 (13.6)	12 (14.6)	0.75
CCI, score	3.27 ± 1.34	3.39 ± 1.50	4.03 ± 1.76	3.95 ± 1.61	0.001
SBP, mmHg	139.8 ± 19.3	144.3 ± 15.7	144.5 ± 15.9	141.1 ± 12.7	0.14
DBP, mmHg	81.9 ± 10.9	81.2 ± 14.3	83.6 ± 12.4	82.7 ± 10.1	0.64
Hemoglobin, g/dL	10.4 ± 1.4	10.2 ± 1.3	10.1 ± 1.5	10.2 ± 1.4	0.72
Albumin, g/L	37.6 ± 4.7	35.6 ± 4.9	35.1 ± 4.9	35.7 ± 4.8	0.01
Hs-CRP, mg/L	1.76 (0.58–3.25)	1.89 (0.68–3.48)	2.39 (1.05–3.88)	2.28 (1.01–3.54)	0.33
rGFR, ml/min/1.73 m^2^	2.55 (0.96–4.52)	2.18 (0.93–4.16)	2.68 (1.12–4.62)	2.45(0.98–4.28)	0.39
FBG, mg/dL	95.8 ± 41.0	101.9 ± 40.0	102.1 ± 44.9	101.5 ± 42.8	0.59
Total Kt/V_urea_, score	2.27 ± 0.56	2.20 ± 0.53	2.10 ± 0.62	2.21 ± 0.51	0.77
WCCr, L/w/1.73 m^2^	69.4 ± 28.0	73.1 ± 29.1	65.1 ± 27.6	66.6 ± 21.9	0.34
Net UF, ml/day	380 (150–580)	400 (200–610)	460 (200–650)	530 (220–700)	0.22
Urine output, ml/day	655 (355–910)	580 (360–830)	620 (390–870)	560 (330–780)	0.32
D/P Cr	0.72 ± 0.12	0.72 ± 0.13	0.71 ± 0.13	0.68 ± 0.12	0.21
PDV/BSA, L/m^2^/day	4.73 ± 0.64	4.90 ± 0.71	4.76 ± 0.78	4.86 ± 0.77	0.41

Data expressed by mean ± standard deviation, number (percent), or median (interquartile range) and p value. Q1: < 0.39 mEq/L; Q2: 0.39 mEq/L to <0.52 mEq/L; Q3: 0.52 mEq/L to <0.69 mEq/L; Q4: ≥0.69 mEq/L. Conversion factors for units: albumin and hemoglobin in g/dL to g/L × 10. FBG in mg/dL to mol/L × 0.05551. No conversion necessary for serum potassium in mEq/L and mmol/L. ESRD, end stage renal disease; CGN, chronic glomerulonephritis; DN, diabetic nephropathy; HN, hypertensive nephrosclerosis; ACEI, angiotensin-converting enzyme inhibitors; ARB, angiotensin II receptor blockades; CCI, Charlson Comorbidity Index; BMI, body mass index; SBP, systolic blood pressure; DBP, diastolic blood pressure; Hs-CRP, high-sensitivity C-reactive protein; FBG, fast blood glucose; rGFR, residual glomerular filtration rate; WCCr, total weekly creatinine clearance; Net UF, net ultrafiltration; D/P Cr, dialysate-to-plasma ratio of creatinine; PDV/BSA, peritoneal dialysis volume per unit of body surface area.

**Table 5:** Logistic regression analysis of risk factors for increased serum potassium fluctuation (standard deviation ≥0.39 mEq/L).

Risk factors	Univariate			Multivariate		
	OR	95% CI	*p*value	OR	95% CI	*p* value
Male	0.931	(0.574–1.510)	0.771	0.909	(0.844–0.979)	0.012
Age	1.017	(1.001–1.033)	0.041			
BMI	0.906	(0.843–0.974)	0.007			
Diabetes status	1.694	(0.818–3.510)	0.156			
CCI	1.245	(1.053–1.472)	0.010	1.223	(1.025–1.460)	0.026
Albumin	0.924	(0.876–0.974)	0.003	0.940	(0.891–0.993)	0.026
Hemoglobin	0.998	(0.987–1.010)	0.785			
Hs-CRP	0.702	(0.397–1.242)	0.224			

Data expressed by OR, 95% CI and p value. OR, odds ratio; CI, confidence interval; BMI, body mass index; CCI, Charlson comorbidity index; Hs-CRP, high-sensitivity C-reactive protein.

**Table 6:** Survival rate for peritoneal dialysis patients by time-averaged serum potassium and its fluctuation.

	t-SP < 4.0 (n = 212)	t-SP ≥ 4.0 (n = 145)	*p* value	t-SP ≥ 4.0		*p*value
				SD < 0.54 (n = 73)	SD ≥ 0.54 (n = 72)	
Overall survival rate						
1 year	0.947 (0.916–0.978)	0.972 (0.945–0.999)	0.262	0.986 (0.959–1.000)	0.958 (0.911–1.000)	0.306
3 years	0.743 (0.678–0.808)	0.880 (0.825–0.935)	0.010	0.942 (0.887–0.997)	0.819 (0.725–0.913)	0.034
5 years	0.516 (0.404–0.628)	0.776 (0.682 − 0.870)	0.001	0.863 (0.745–0.981)	0.701 (0.566–0.836)	0.018
Cardiovascular survival rate						
1 year	0.980 (0.960–1.000)	0.979 (0.955–1.000)	0.915	0.986 (0.959–1.000)	0.971 (0.932–1.000 )	0.549
3 years	0.849 (0.792–0.906)	0.923 (0.876–0.970)	0.122	0.972 (0.933–1.000)	0.873 (0.789–0.957)	0.047
5 years	0.656 (0.536–0.776)	0.844 (0.756–0.932)	0.028	0.923 (0.823–1.000)	0.771 (0.640–0.902)	0.019

Data expressed by rate (95% CI) and p value. t-SP, time-averaged serum potassium (mEq/L); SD, standard deviation (mEq/L).

## References

[b1] Musso C. G. Potassium metabolism in patients with chronic kidney disease. Part II: patients on dialysis (stage 5). Int Urol Nephrol 36, 469–72 (2000). 15783126 10.1007/s11255-004-6194-y

[b2] Chuang Y. W. *et al.* Hypokalaemia: an independent risk factor of Enterobacteriaceae peritonitis in CAPD patients. Nephrol Dial Transplant 24, 1603–8 (2009). 19103738 10.1093/ndt/gfn709

[b3] Szeto C. C. *et al.* Hypokalemia in Chinese peritoneal dialysis patients: prevalence and prognostic implication. Am J Kidney Dis 46, 128–35 (2005). 15983966 10.1053/j.ajkd.2005.03.015

[b4] Torlen K. *et al.* Serum potassium and cause-specific mortality in a large peritoneal dialysis cohort. Clin J Am Soc Nephrol 7, 1272–84 (2012). 22626960 10.2215/CJN.00960112 PMC3408121

[b5] Kwan B. C. & Szeto C. C. Dialysis: Hypokalaemia and cardiac risk in peritoneal dialysis patients. Nat Rev Nephrol 8, 501–3 (2012). 22801950 10.1038/nrneph.2012.159

[b6] Xu Q. *et al.* Serum potassium levels and its variability in incident peritoneal dialysis patients: associations with mortality. PloS one 9, e86750 (2014). 24475176 10.1371/journal.pone.0086750 PMC3903570

[b7] Khan A. N., Bernardini J., Johnston J. R. & Piraino, B. Hypokalemia in peritoneal dialysis patients. Perit Dial Int 16, 652 (1996). 8981546

[b8] Tziviskou E. *et al.* Prevalence and pathogenesis of hypokalemia in patients on chronic peritoneal dialysis one center’s experience and review of the literature. Int Urol Nephrol 35, 429–34 (2003). 15160552 10.1023/b:urol.0000022867.93739.03

[b9] Zanger R. Hyponatremia and hypokalemia in patients on peritoneal dialysis. Seminars in dialysis 23, 575–80 (2010). 21121953 10.1111/j.1525-139X.2010.00789.x

[b10] Spital A. & Sterns R. H. Potassium supplementation via the dialysate in continuous ambulatory peritoneal dialysis. Am J Kidney Dis 6, 173–6 (1985). 3898826 10.1016/s0272-6386(85)80022-6

[b11] Clausen T. & Everts M. E. Regulation of the Na, K-pump in skeletal muscle. Kidney Int 35, 1–13 (1989). 2540370 10.1038/ki.1989.1

[b12] Hundal H. S. *et al.* Insulin induces translocation of the alpha 2 and beta 1 subunits of the na+/k(+)-atpase from intracellular compartments to the plasma membrane in mammalian skeletal muscle. J Biol Chem 267, 5040–3 (1992). 1312081

[b13] Tzamaloukas A. H. & Avasthi P. S. Temporal profile of serum potassium concentration in nondiabetic and diabetic outpatients on chronic dialysis. Am J Nephrol 7, 101–9 (1987). 3605230 10.1159/000167443

[b14] Sica D. A. Importance of Potassium in Cardiovascular Disease. J Clin Hypertens (Greenwich) 4, 198–206 (2002). 12045369 10.1111/j.1524-6175.2002.01728.x PMC8101903

[b15] Oikawa K. *et al.* Prognostic value of heart rate variability in patients with renal failure on hemodialysis. Int J Cardiol 131, 370–7 (2009). 18199499 10.1016/j.ijcard.2007.10.033

[b16] Di Iorio B. *et al.* Blood pressure variability and outcomes in chronic kidney disease. Nephrol Dial Transplant 27, 4404–10 (2012). 22962409 10.1093/ndt/gfs328

[b17] Kainz A., Mayer B., Kramar R. & Oberbauer R. Association of ESA hypo-responsiveness and haemoglobin variability with mortality in haemodialysis patients. Nephrol Dial Transplant 25, 3701–6 (2010). 20507852 10.1093/ndt/gfq287 PMC3360143

[b18] Kalantar-Zadeh K. *et al.* Survival predictability of time-varying indicators of bone disease in maintenance hemodialysis patients. Kidney Int 70, 771–80 (2006). 16820797 10.1038/sj.ki.5001514

[b19] Kim H. W. *et al.* Factors associated with hypokalemia in continuous ambulatory peritoneal dialysis patients. Electrolyte & blood pressure 5, 102–10 (2007). 24459508 10.5049/EBP.2007.5.2.102 PMC3894509

[b20] Mehrotra R. *et al.* Serum albumin as a predictor of mortality in peritoneal dialysis: comparisons with hemodialysis. Am J Kidney Dis 58, 418–28 (2011). 21601335 10.1053/j.ajkd.2011.03.018 PMC3159826

[b21] Struijk D. G. *et al.* The effect of serum albumin at the start of continuous ambulatory peritoneal dialysis treatment on patient survival. Perit Dial Int 14, 121–6 (1994). 8043663

[b22] Lowrie E. G., Huang W. H. & Lew N. L. Death risk predictors among peritoneal dialysis and hemodialysis patients: a preliminary comparison. Am J Kidney Dis 26, 220–8 (1995). 7611256 10.1016/0272-6386(95)90177-9

[b23] Beddhu S. *et al.* The effects of comorbid conditions on the outcomes of patients undergoing peritoneal dialysis. Am J Med 112, 696–701 (2002). 12079709 10.1016/s0002-9343(02)01097-5

[b24] Descoeudres B. *et al.* Contribution of early failure to outcome on peritoneal dialysis. Perit Dial Int 28, 259–67 (2008). 18474918

[b25] Rocco M. V., Jordan J. R. & Burkart J. M. Determination of peritoneal transport characteristics with 24-hour dialysate collections: dialysis adequacy and transport test. J Am Soc Nephrol 5, 1333–8 (1994). 7893998 10.1681/ASN.V561333

[b26] Konings C. J. *et al.* Fluid status in CAPD patients is related to peritoneal transport and residual renal function: evidence from a longitudinal study. Nephrol Dial Transplant 18, 797–803 (2003). 12637651 10.1093/ndt/gfg147

[b27] Van Olden R. W., Krediet R. T., Struijk D. G. & Arisz L. Measurement of residual renal function in patients treated with continuous ambulatory peritoneal dialysis. J Am Soc Nephrol 7, 745–50 (1996). 8738810 10.1681/ASN.V75745

[b28] Wang A. Y. *et al.* Cardiac valve calcification as an important predictor for all-cause mortality and cardiovascular mortality in long-term peritoneal dialysis patients: a prospective study. J Am Soc Nephrol 14, 159–68 (2003). 12506148 10.1097/01.asn.0000038685.95946.83

[b29] Chertow G. M. *et al.* Effect of cinacalcet on cardiovascular disease in patients undergoing dialysis. N Engl J Med 367, 2482–94 (2012). 23121374 10.1056/NEJMoa1205624

